# Sales promotion in health and medicine: using incentives to stimulate patient interest and attention

**DOI:** 10.1186/s12913-020-05601-y

**Published:** 2020-09-15

**Authors:** James K. Elrod, John L. Fortenberry

**Affiliations:** 1 Willis-Knighton Health System, 2600 Greenwood Road, Shreveport, LA 71103 USA; 2 LSU Shreveport, 1 University Place, Shreveport, LA 71115 USA

**Keywords:** Sales promotion, Marketing communications, Promotion, Hospitals, Healthcare

## Abstract

**Background:**

Sales promotion—the use of incentives to encourage patronage—is a staple of marketing communications in the health services industry. Sales promotion applications commonly used by health services organizations include free samples, free trials, coupons, contests, and loyalty programs. These avenues engender goodwill, appreciation, and attentiveness; they also serve as small, but powerful promotional mechanisms by reminding recipients of healthcare institutions, compelling particular actions, encouraging repeat business, or prompting some related desirable in an effort to hasten exchange and bolster loyalty.

**Discussion:**

Sales promotion offers myriad opportunities for healthcare providers to connect with audiences. While limited in their power to attract broad audiences when used in isolation, sales promotion avenues used in tandem with other marketing communications create helpful engagement synergies which amplify conveyance initiatives. This article presents an overview of sales promotion and notably shares deployment insights and experiences from Willis-Knighton Health System, permitting peer healthcare establishments to view associated pathways, reflect on their own sales promotion efforts, and potentially bolster initiatives with the perspectives supplied herein.

**Conclusions:**

Sales promotion offers healthcare providers a complementary communications avenue, helping to reinforce other elements of the marketing communications mix, affording opportunities to develop better connections with patients. In formulating associated communication plans, health and medical establishments should be reminded of the benefits offered by sales promotion and especially strive to effect creative applications that build interest and attention. By doing so, opportunities to bolster patient volume and increase all-important market share abound.

## Background

Health and medical institutions exist to serve [1–3], and in doing so, they obviously must proficiently attract and retain patients [4–9]. With patient acquisition and retention being vital determinants of success, associated skills must be shaped, honed, and continually developed over the life cycles of healthcare establishments, with communications acumen being one of greatest importance [5, 9–14]. Healthcare institutions direct communications toward current and prospective patients in many ways, typically selecting one or more established methods from the discipline of marketing. One of these methods is known as sales promotion, an element of what is known as the marketing communications mix, a 5-component array which also includes advertising, personal selling, public relations, and direct marketing [5, 11, 15]. Sales promotion involves the use of incentives, such as contests and free giveaways, to encourage patronage [5, 15]. Known for its ability to generate interest and attention, sales promotion is a staple of marketing communications in the health services industry, with the component being deployed in a wide variety of fashions [5, 14, 16–18].

A typical sales promotion application in health and medical institutions involves the distribution of free gifts, such as pens, calendars, magnets, paperweights, and similar items—usually bearing the logos of given establishments—to current and prospective patients and other publics of healthcare facilities. Other sales promotion applications include free samples, free trials, coupons, contests, and loyalty programs. These items engender goodwill, appreciation, and attentiveness; they also serve as small, but powerful promotional mechanisms by reminding recipients of given institutions, compelling particular actions, encouraging repeat business, or prompting some related desirable in an effort to hasten exchange and bolster loyalty [5, 14, 16–20].

Sales promotion efforts usefully complement other elements of the marketing communications mix, creating helpful synergies which reinforce the aggregate communicative efforts of institutions [17–19, 21]. Communicative avenues making use of sales promotion indeed are heavily traveled by healthcare establishments, placing a premium on developing highly creative applications which foster differentiation, enhance memorability, and elevate interest and attention. Given the broad range of sales promotion possibilities, especially hastened by creative influences, benefits abound whenever opportunities arise to examine the associated efforts of peer healthcare institutions. This article presents one such opportunity, sharing sales promotion insights and experiences from Willis-Knighton Health System, permitting peer healthcare establishments to view associated pathways, reflect on their own sales promotion efforts, and potentially bolster initiatives with the perspectives supplied herein.

## Definition and overview

Sales promotion is one of many elements constituting the broad discipline of marketing, formally defined as “a management process that involves the assessment of customer wants and needs, and the performance of all activities associated with the development, pricing, provision, and promotion of product solutions that satisfy those wants and needs” [5], p. 288. Promotion, as evidenced in this definition, is a core feature of marketing, earning inclusion as one of the Ps in the classic expression known as the *four Ps of marketing* (i.e., Product, Price, Place, Promotion). The promotion aspect of marketing essentially entails any and all elements associated with engaging audiences, with the core pathways for engagement being depicted in a descriptive model known as the marketing communications (or promotions) mix [4, 5].

The marketing communications mix, as traditionally depicted, contains five principal avenues of communication; namely, advertising (i.e., the paid use of mass media to deliver messages), personal selling (i.e., the use of sales agents to personally deliver messages), sales promotion (i.e., the use of incentives, such as contests and free giveaways, to encourage patronage), public relations (i.e., the use of publicity and other unpaid promotional methods to deliver messages), and direct marketing (i.e., the delivery of messages via mail, the Internet, and similar routes directly to consumers) [5, 11]. Healthcare providers examine each of these communicative avenues, selecting one or more believed to be most capable of reaching target audiences, with the ultimate goal being to encourage patronage or compel some other desired action [5, 15].

Sales promotion is a unique aspect of the marketing communications mix, relying on incentives to encourage patronage [5, 15]. Examples of sales promotion as deployed by health and medical institutions include the following.

- Free gifts: A medical center’s birthing unit delivers gift baskets to new mothers; a cosmetic surgery clinic distributes logo-bearing skin care products to patients at the conclusion of their visits.
- Free trials: A hospital’s wellness center offers free 30-day trial memberships, permitting prospects to evaluate the facility and its services; a weight loss clinic offers prospects opportunities to partake in 3-month trials to evaluate a special diet program.
- Coupons: An eye clinic offers a coupon entitling recipients to a $500 discount for laser vision correction surgery; a dental clinic offers a coupon granting holders a 10% discount which can be applied toward any dental service.
- Contests: A nursing home offers residents or their responsible parties the opportunity to be entered into a cash prize draw in return for participating in a customer satisfaction survey; a pediatric medical center offers patients the opportunity to participate in an art contest with the winning submission being placed on permanent display.
- Loyalty programs: A hospital offers a patient VIP program, affording access to a range of gratuities, including covered parking, free food in the cafeteria, a monthly newsletter, and more; a dental practice offers patients a free dental cleaning service after every 10 dental cleaning services received.

The expense associated with selected incentives varies, but the items or opportunities tend to be of negligible value in light of benefits received or expected. They very often are intended to serve as small tokens of appreciation awarded for engaging in some desired action (e.g., visiting a clinic, participating in a survey, being a loyal customer), achieving something momentous (e.g., a health or wellness milestone), reaching a celebratory point (e.g., a birthday or anniversary), and the like. At other times, they are deployed to compel those not associated with given healthcare establishments to become patrons. Depending on aims, incentives can be directed toward current or prospective patients (i.e., business-to-consumer applications) or health and medical providers (i.e., business-to-business applications), with an excellent example of the latter being distribution of logo-bearing pens, baked goods, or similar items to patient referral sources in the community, demonstrating appreciation for past referrals or perhaps setting the stage for future referrals. Regardless of form or target, sales promotion applications ultimately seek to stimulate the interest and attention of desired audiences [5, 9–11, 17–19].

Sales promotion, used in isolation, cannot reach audiences of the size achieved by other components of the marketing communications mix, especially those that use mass media channels, such as advertising. But these barriers can be traversed by combining sales promotion pursuits with other marketing communications elements to create conveyance synergies. A medical clinic’s television advertisement, for example, could incorporate a reference that new patients will receive a free health planner—a sales promotion element—when they present at the clinic, amplifying the availability of the incentive far beyond that achieved merely by onsite conveyances. A direct mail piece marketing an eye clinic could include a 50% off coupon which could be applied toward the purchase of prescription lenses, combining direct marketing and sales promotion. These decisions, of course, are dependent on the desires of given healthcare establishments, as echoing the availability of incentives via mass media or other channels of communication certainly would be expected to hasten the number of individuals taking advantage of the opportunities, increasing associated costs. Ultimately, with sales promotion, the depth and breadth of distribution can be fine-tuned to meet designated engagement goals and objectives, affording myriad conveyance options [5, 11, 17–21].

## Institutional background, deployment history, and context within marketing communications

From its earliest of days, dating back to 1924, Willis-Knighton Health System has emphasized communications excellence, something which in present times remains a strategic priority, compelling extensive communicative experimentation and innovation [4, 7, 22]. Headquartered in Shreveport, Louisiana and situated in the heart of an area known as the Ark-La-Tex where the states of Arkansas, Louisiana, and Texas converge, Willis-Knighton Health System holds market leadership in its served region where it delivers comprehensive health and wellness services through multiple hospitals, numerous general and specialty medical clinics, an all-inclusive retirement community, and more. The system’s achievement of market leadership is attributed, in part, to communications prowess, permitting Willis-Knighton Health System to effectively engage current and prospective patients, evoking interest and attention, ultimately leading to burgeoning patient volume and customer loyalty.

Today, Willis-Knighton Health System leverages the power of the full marketing communications mix, deploying all of its components, including sales promotion. The institution’s use of sales promotion is historic, dating back to its origins. Early sales promotion efforts would be considered to be primitive by modern standards. Indeed, the days of simple calendars marked with block text depicting the name, address, and telephone number of the institution or basic ink pens imprinted with a similar plain presentation are things of the past. Today, thanks to vastly improved production processes, highly innovative concepts, advanced understanding of promotional avenues, and more, sales promotion efforts deployed by Willis-Knighton Health System are more impressive than ever.

**Fig. 1:**
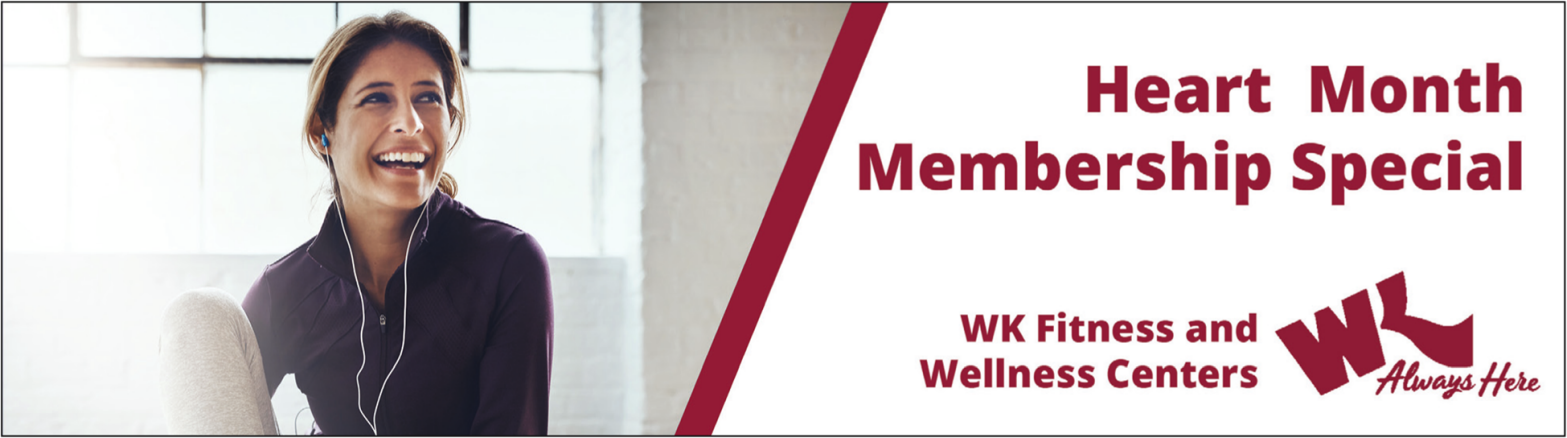
A billboard advertisement communicating a sales promotion offered by WK Fitness and Wellness Centers

In recent years, Willis-Knighton Health System has used a wide range of items for its various sales promotion endeavors. Its most frequently utilized endeavors center on the distribution of free gifts (e.g., logo-bearing pens, memo pads, first aid kits), with most of them being circulated as simple gestures of gratitude, primarily aimed at fostering loyalty among current patients, as opposed to building patient volume. Beyond free gifts, the institution has offered discounts to individuals who attend particular seminars, such as those profiling laser vision correction services. Willis-Knighton Health System’s Fitness and Wellness Centers have offered a range of enticements, including membership specials and similar incentives, as Fig. 1—a billboard advertisement conveying an associated sales promotion—demonstrates. The Oaks of Louisiana, Willis-Knighton Health System’s senior living community, has offered move-in specials as an incentive for individuals to become assisted living residents.

**Fig. 2:**
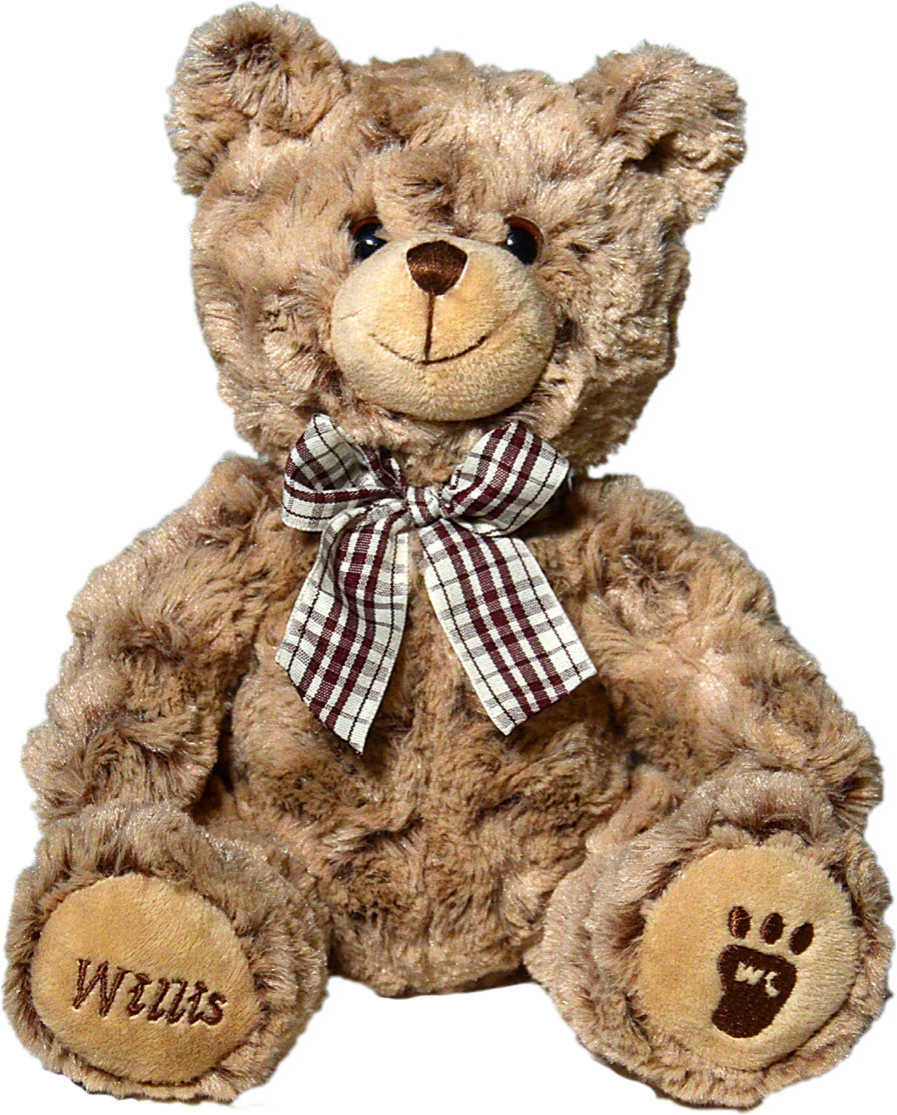
Willis-Knighton Health System’s Willis the Bear

**Fig. 3:**
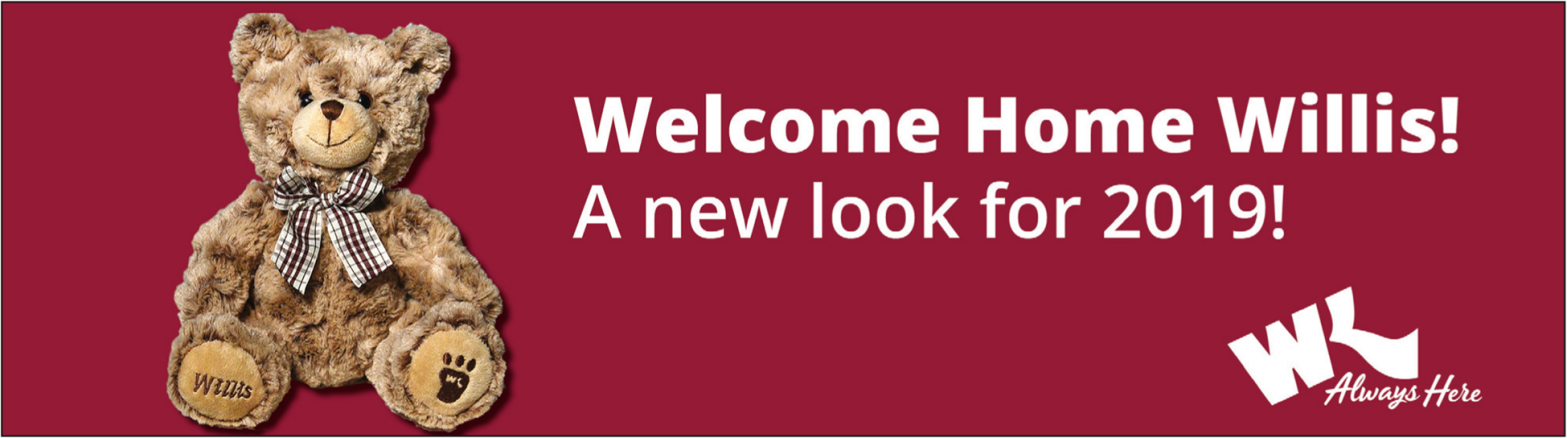
A billboard advertisement featuring Willis-Knighton Health System’s Willis the Bear

But the most notable of sales promotion efforts forwarded by Willis-Knighton Health System is that of its Willis the Bear mascot, presented in Fig. 2, which is distributed exclusively to women who give birth at a Willis-Knighton Health System facility. This particular item was developed from the ground up, with executives working directly with a teddy bear manufacturer to create a custom stuffed animal to represent Willis-Knighton Health System and its maternity services offering. While developing Willis the Bear carried far greater costs than the typical sales promotion application which utilizes prefabricated items, its associated value to the institution has more than covered the expenditures, generating extensive publicity and offering myriad marketing communications opportunities beyond sales promotion, as Fig. 3—a billboard advertisement featuring Willis the Bear—demonstrates. Indeed, Willis the Bear is the preeminent sales promotion effort offered by the institution [22, 23].

While constituting a relatively small part of Willis-Knighton Health System’s overall marketing communications budget, sales promotion efforts are viewed to be valuable endeavors, offering another method to bolster interest and attention in the quest to connect with patients and increase opportunities to serve. Less recognized as a marketing communications pathway than other elements of the marketing communications mix, such as advertising and direct marketing, sales promotion is capable of playing a critical role in advancing the conveyance goals of health services organizations.

## Strengths

With nearly one century of associated experience, Willis-Knighton Health System’s sales promotion efforts have evolved over the decades, as both the institution and available incentives have evolved, with continued use being warranted to present day. A number of strengths of sales promotion have particularly motivated persistency of use, with these characteristics being described as follows.

### Potential to stimulate interest and attention

Sales promotion is well known for its ability to stimulate interest and attention, with this often being cited as its greatest strength. Evoking interest and attention sets the stage for the target audiences of sales promotion efforts to extend their patronage, facilitating the all-important marketing goal of exchange, leading to the capture of market share. Of course, the nature of sales promotion campaigns will dramatically impact the degree of interest and attention generated, making deployment characteristics (e.g., selection of incentives, timing of promotions, methods of presentation) very important [5, 9–11, 17, 18].

A logo-bearing ink pen handed out to the patients of a dermatology clinic likely will be appreciated and serve as a useful reminder by those receiving such, but it will not generate the kind of interest and attention that more sophisticated sales promotion incentives can elicit. Consider, however, a discount coupon for laser vision correction services placed in an eye clinic’s advertisement, an online advertisement offering a chance to win an all-expense paid trip to a major sporting event in exchange for attending a sports medicine clinic’s seminar on the latest orthopedic surgery techniques, or a direct mail parcel noting that all who attend a heart institute’s grand opening will receive a logo-bearing fleece blanket. These would constitute more advanced incentives, with the examples also merging associated incentives with other forms of marketing communication to magnify promotional impact. As with most any marketing pursuit, sales promotion efforts require institutions to stipulate desired goals and make selections, accordingly, to achieve those particular desires.

### Ability to synergize communications

Sales promotion serves as an excellent complement to other forms of marketing communication. When used in tandem with other methods, synergies are created that extend the communicative impact of given promotions. In the examples presented in the preceding paragraph (i.e., the eye clinic, the sports medicine clinic, the heart institute), inclusion of sales promotion incentives in the given communications (i.e., the advertisements, the direct mail piece) likely will generate much greater attentiveness toward those communications. In some respects, inclusion of a sales promotion incentive can help to paint a bullseye on advertisements, direct mail pieces, and other forms of communication, drawing eyes that would otherwise be disinterested or perhaps too occupied to notice given conveyances. In such cases, incorporating sales promotion incentives into other forms of communication magnifies the value of those particular overtures [16–21].

Of course, care must be taken not to overutilize sales promotion as a communicative mechanism, as doing so can diminish the special nature of given promotions, making them common and expected, negatively impacting its primary attribute of generating interest and attention. In some circumstances, such as that of couponing, overuse can stall patronage whenever these promotions are withdrawn, as potential patients will simply wait for given coupons to appear again before pursuing services. That said, sales promotions should be considered as special offers available for a limited time after which they are withdrawn for undefined periods, ensuring that they indeed remain special and retain their compelling effects.

### Opportunities for vast customization

Options abound with sales promotion, courtesy of many different forms (e.g., free gifts, coupons, discounts, contests), conveyance partners (e.g., other elements of the marketing communications mix), circulation plans (e.g., timing and length of promotion), and other deployment aspects. Typical applications can be observed across most any market, but healthcare institutions certainly should not feel limited to such. Sales promotion permits extreme latitude, as it essentially entails the assembly of incentives viewed to be attractive and compelling to target audiences. The possibilities for such are endless, limited only by the creative imagination and, of course, budgetary constraints [5, 9–11, 14, 17–21].

Additional expenditures normally will be required to realize highly creative applications, but greater returns on those particular investments should be expected, as Willis-Knighton Health System experienced with its Willis the Bear mascot. The greater the differentiation from typical applications, the more likely interest and attention will be bolstered, permitting given sales promotion efforts to excel. It certainly is worth experimenting with things that defy the sales promotion norms of given marketplaces. With the component being somewhat boundless, imaginative perspectives are welcomed and just might lead to something truly novel, boosting marketing communications.

## Limitations

While motivations for using sales promotion are compelling, it is vital to understand associated limitations as they must be factored into deployments. Fortunately, by gaining an awareness of potential obstacles followed by prudent planning and implementation, barriers can be traversed effectively. Notable limitations are described as follows.

### Limited impact in isolation

Sales promotion as a communications and engagement mechanism is not well suited for use in isolation. A coupon offering a discounted membership at a hospital’s wellness center, for example, is of limited value without being piggybacked onto other communications platforms. Onsite coupon distribution, while representing a nice gesture that likely would be appreciated, is not particularly compelling, as prospects are already onsite and potentially ready to purchase memberships, even without an enticement. However, if the coupon is placed in an advertisement in a local newspaper, forwarded via a direct mail piece to area residents, or placed in the hands of a wellness center representative who distributes them as community contacts are made, then generation of significant interest and attention becomes a distinct possibility.

There certainly are situations where limited use of sales promotion incentives is desirable, as in cases where the goal is simply to issue small tokens of appreciation to customers, calling for limited distribution of, say, a logo-bearing first aid kit to patients of an urgent care center. Certainly such an approach would be justified in cases where patient retention, as opposed to patient acquisition, is the primary goal. Regardless, healthcare providers considering the use of sales promotion as a conveyance mechanism must remain aware of its limited potential when used independently of other communications. As a complementary communicative tool, tandem deployments which incorporate other elements of the marketing communications mix generally are advised [16–21].

### Escalates communications expenditures

Quite obviously, the incentives featured in sales promotion applications carry costs, with level of expenditure predicated by the type of item offered, degree of distribution, duration of use, and related variables impacting associated financial outlays. Regardless of selections, whether bargain basement or otherwise, such pursuits increase the aggregate marketing communications costs associated with promoting healthcare services, especially if sales promotion is used to its fullest as a complementary element to other conveyances [17–21]. Any administrative pursuit, particularly those requiring financial outlays, requires prudent planning as this can aid in ensuring that time, money, and other necessary resources are deployed in a manner to increase the likelihood of achieving desired results. Sales promotion as a communicative mechanism should be treated in the same manner. As long as sales promotion efforts are well devised and implemented, healthcare institutions should remain at least cautiously optimistic that associated resource requirements will generate an acceptable return on investment. When viewing sales promotion in this light, the pathway should be considered an investment directed toward the advancement of communications goals.

### Potential to be replicated by competitors

Most any incentive used in sales promotion applications can be replicated, making this particular pathway a difficult one in which to achieve a competitive advantage. Commonly deployed free gifts, such as pens, notepads, calendars, and the like, are usually sourced by one of many promotional products companies, which offer similar, if not identical, offerings to anyone willing to make a purchase. Coupons for discounted services can be matched—or even surpassed—with relative ease by competitors. The same could be said of contests, loyalty programs, and other types of incentives [5, 11, 16–21]. Perhaps the best workaround in defense of replication involves the identification of highly creative, unique incentives, with these attributes diminishing the ability of competitors to follow suit and mirror such. The resources required to realize a high-profile sales promotion incentive almost certainly will be intensive, but the prospect of gaining a promotional offering that incorporates natural defenses against competitive replication is quite compelling and a worthy reward for pursuing this laborious pathway.

## Operational reflections

For administering any component of the marketing communications mix, Willis-Knighton Health System advises establishing a baseline foundation of resources, including (1) top leadership support and commitment, (2) financial resources sufficient for funding communications activities, (3) competent personnel charged with effecting given initiatives, and (4) formal processes permitting effective planning, implementation, and evaluation of initiatives. Adequate resources set the stage for productive audience engagement endeavors, minimizing chances of resource-depleting and reputation-damaging mistakes which, in the realm of marketing communications, often are very public, given the open circulation of such conveyances. These resources also ensure competencies in using given marketing communications mix components, with proper deployment being essential for realizing desired outcomes.

Beyond the advisories conveyed elsewhere in this article, Willis-Knighton Health System suggests that healthcare institutions desirous of deploying sales promotion applications develop at least one high-profile incentive, something highly creative and unique which garners extremely high interest and attention and is less likely to be copied by competitors due to its distinctiveness. In Willis-Knighton Health System’s case, Willis the Bear serves as its high-profile incentive, anchoring its sales promotion program. While it would be nice for all sales promotion incentives to be highly distinctive, for most institutions, the resource requirements associated with doing so invariably will be excessive. But temporal and financial resources for developing, say, one or two high-profile items might possibly be available and, if so, investment in such can prove highly beneficial. This sort of pursuit will stimulate broad sales promotion efforts, ensuring that at least some applications go beyond routine deployments, helping to achieve a form of competitive advantage.

Willis-Knighton Health System has observed another occurrence for which healthcare institutions involved in sales promotion should be on guard. Free gifts, if not closely monitored, can get out of hand very quickly without disciplined ordering processes and inventory control systems. Without effective protocols, wasteful scenarios abound, including multiple orders, excessive inventory, inventory shrinkage, and related resource drains. Even though the per-item price of many promotional products is negligible, aggregate costs across an institution, especially large systems, can add up very quickly. If deployed effectively, associated financial outlays can be considered to be investments, but if mismanaged, such outlays become nothing more than losses, wasting precious resources. A disciplined approach is the best defense against such waste, permitting sales promotion efforts the greatest opportunity possible to make a positive contribution to the communicative goals of healthcare institutions.

## Conclusions

Sales promotion offers healthcare providers a complementary communications avenue, helping to reinforce other elements of the marketing communications mix, permitting associated synergies and opportunities to develop better connections with current and prospective patients. In formulating communications plans, health and medical providers should be reminded of the benefits afforded by sales promotion, incorporating it, when and where possible, for purposes of enriching conveyance pursuits, with a special eye toward effecting highly creative applications to amplify interest and attention. As evidenced by Willis-Knighton Health System’s deployment experiences, sales promotion affords unique opportunities to bolster patient volume and grow vital market share, permitting healthcare providers increasing opportunities to demonstrate their proficiencies in service to others.

## Data Availability

Not applicable.
